# The ability of waist-to-height ratio to identify health risk

**DOI:** 10.11606/s1518-8787.2019053000895

**Published:** 2019-09-12

**Authors:** Márcia Mara Corrêa, Luiz Augusto Facchini, Elaine Thumé, Elizabete Regina Araújo de Oliveira, Elaine Tomasi

**Affiliations:** I Universidade Federal de Pelotas. Programa de Pós-Graduação em Epidemiologia. Pelotas, RS, Brasil; II Universidade Federal de Pelotas. Programa de Pós-Graduação em Enfermagem. Pelotas, RS, Brasil; III Universidade Federal do Espírito Santo. Programa de Pós-Graduação em Saúde Coletiva. Vitória, ES, Brasil

**Keywords:** Body Mass Index, Waist Circumference, Body Height, Overweight, Risk Factors, Hypertension, diagnosis

## Abstract

**OBJECTIVE:**

To evaluate the performance of the ratio between the waist circumference and the height in the identification of health risk compared with the correlation matrix between the anthropometric parameters body mass index and waist circumference.

**METHODS:**

A population-based study presenting a transversal cut in a representative sample of the Brazilian adult and older population. The combination of the body mass index with the waist circumference resulted in health risk categories, and the cutoff points of the ratio between the waist circumference and the height as anthropometric indicator were used for classification of low and increased risk. Poisson regression was used to verify the association of systemic arterial hypertension with the health risk categories.

**RESULTS:**

The results showed 26% of adult men, 10.4% of adult women and more than 30% of the older adults of both genders classified as without risk by the combination matrix between body mass index and waist circumference presented a ratio between the waist circumference and height that showed increased risk. All risk categories continued to be associated with hypertension after control for confounding factors, being almost two times higher for adults with moderate and high risk according to both methods. When the waist-to-height ratio was used as a risk indicator, the prevalence of hypertension ratios for the older adults was 1.37 (95%CI 1.16–1.63) and 1.35 (95%CI 1.12–1.62) for men and women, respectively, being these values close to the combination matrix body mass index and waist circumference.

**CONCLUSIONS:**

The waist-to-height ratio identified more individuals at early health risk than the combination matrix between the body mass index and the waist circumference and showed comparable ability to identify health risk, regardless of gender and age, regarding the prevalence ratios for systemic arterial hypertension.

## INTRODUCTION

Obesity and, more recently, overweight have been recognized as major public health problems in many countries^1^, including Brazil^2^, and several attempts have been made to identify the best anthropometric predictor^3^ for several non-communicable diseases and complications in different populations and age groups. For overweight diagnosis, several techniques have been proposed to accurately estimate the total amount of body fat, as well as its distribution^4^. Considering the costs of this method and its easy use, both in epidemiological studies and in the clinical practice, the use of body mass index (BMI)^1^ and the waist circumference (WC)^1^ as effective anthropometric indicators has been proposed in this type of evaluation.

As the relationship between BMI and the risk of morbidities can be affected by the body fat distribution, regardless of the body weight^5^, studies have recommended the combination of this index with other measurements of abdominal adiposity, with emphasis on WC, for a better diagnosis of overweight as a health risk predictor^6^. Excess abdominal fat has been associated with disorders in glucose and lipid metabolism, which relate to cardiovascular diseases, insulin resistance, and systemic arterial hypertension (SAH)^9^. For these reasons, WC has been recommended as a cardiometabolic risk anthropometric marker^6^.

However, a recent study suggests the use of the ratio between waist circumference and height (WHR) as a substitute anthropometric measure for the correlation matrix between BMI and WC because of its greater ability to identify individuals at health risk, in addition to being strongly associated with cardiovascular and metabolic risk factors, regardless of body weight^11^. A systematic review and meta-analysis conducted in 2012 with more than 300 thousand individuals concluded that WHR is the best screening tool to detect cardiometabolic risk factors in both genders and in several ethnic groups, showing its superiority over BMI and WC^12^.

The use of a simple, easy-to-interpret and low-cost measure in epidemiological studies and in the clinical practice, both individual and collective, especially which can be performed as a screening method in programs for health promotion and prevention of non-communicable diseases and complications – incited the conduction of this study. The objective was to evaluate the ability of WHR to identify health risks, especially SAH, compared with the correlation matrix between BMI and WC anthropometric indicators. In Brazil, no population-based study using WHR as an anthropometric indicator of health risk in a representative sample of the adult and older population was published by now.

## METHODS

This study is part of a population-based epidemiological survey, carried out in 2008 and 2009, which aimed to evaluate the access to and the quality of care in the health system in residents of urban areas of 100 municipalities in the 23 Brazilian states. This is a cross-sectional, population-based study in a representative sample of the Brazilian adult and older population, and individuals hospitalized, legally deprived of liberty, or residing in long-term institutions were considered ineligible for the study. The survey consisted of a total sample of 13,756 adults and 7,015 older individuals. The percentage of losses and refusals was, respectively, 8% and 2% for the adult population and 4% and 2% for the older population. Key informant interviews, without anthropometric data (3,998 adults and 1,128 older adults), were excluded, thus obtaining a final sample of 8,235 adults and 5,494 older adults with the anthropometric measurements necessary for the analysis. This sample showed statistical power higher than 95% for the correlations tested.

For the selection of municipalities and urban census tracts, data from the 2000 Brazilian Population Census, carried out by the Brazilian Institute of Geography and Statistics (IBGE)^13^, were used. The spatial and population reference standard used for sample estimates was the urban census tract, defined as an aggregate of about 300 households and 1,000 inhabitants, and the municipalities were grouped by population size, being denominated: “very small” those with less than 10,000 inhabitants, “small” those from 10,000 up to 20,000 inhabitants, “medium” those from 20,000 up to 100,000 inhabitants, “large” those from 100,000 up to 1.1 million inhabitants, and “very large” those from 1.1 million inhabitants on. In each municipality, census tracts were randomly selected, and in each of them, independent samples of adults and older adults were identified. For the samples with adults, 10 households were visited, and for the older adults, 30 households, following a systematic “jump” between the houses. Using this strategy, the expectation was to interview about 19 adults and 10 older adults per sector. All eligible individuals were included in each household, even if the predefined quota was exceeded.

Data were collected by duly trained research assistants, and the questionnaires were divided into five blocks: identification, health promotion and preventive care, health problems, access to and use of health services, and anthropometric measurements. The questionnaires were available in a palmtop computer (personal digital assistant or PDA).

Using the techniques proposed by Lohman et al.^14^, the anthropometric variables weight, height and WC were measured twice, and the final result of each variable was obtained by calculating the arithmetic mean. WC was obtained between the iliac crest and the lateral costal margin (midpoint between the hip and the last rib) with a precision of 0.1 cm.

The values of WC were considered for the diagnosis of abdominal fat accumulation, and values lower than 80 and 94 cm were classified as low accumulation for women and men, respectively. Values between 94 and 102 cm for men and between 80 and 88 cm for women were classified as high accumulation; values above 102 cm for men and above 88 cm for women, as very high^1^.

**Figure 1:**
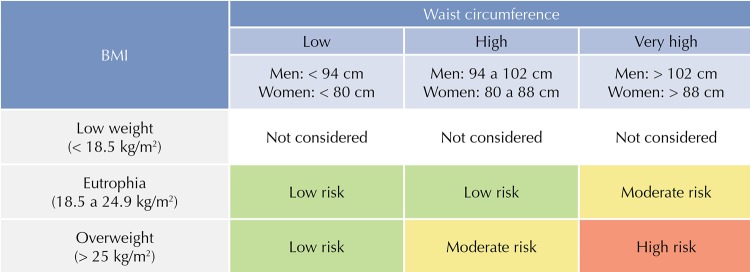
Health risk categories using the correlation matrix between BMI and the waist circumference.

By dividing the body weight (kg) by the height (m) squared (P/A^2^), BMI was calculated, whose classification was based on the standard proposed by the World Health Organization (WHO)^1^. According to Molarius et al.^15^, the association of BMI measurement with WC offers a combination for health risk assessment, in addition to decreasing the limitations of the isolated use of each of the measurements. The correlation matrix of these measurements resulted in the categories of health risk, as shown in Figure 1.

BMI: body mass index

WHR was calculated using the measurement of WC divided by height, both in centimeters (cm), and the maximum result of the equation was equal to one. In Brazil, a recent population-based study set the value of 0.55 as the cutoff point of WHR as an anthropometric indicator of overweight in older adults^16^, using BMI as an anthropometric reference. However, a reference cutoff value of WHR as a marker of overweight for adults was not found in the national literature; therefore, it was established using the ROC curve (receiver operating characteristic). The area under the ROC curve (AUROC) and the cutoff points of WHR with higher sensitivity and specificity values were used as criteria to identify overweight, using BMI as an anthropometric reference. The cutoff point 0.50, both for adults and for older adults, was used to classify low and increased risk as health risk categories.

Blood pressure (BP) was measured using a digital automatic wrist-cuff device, and two measurements were performed with a minimum interval of fifteen minutes between them, following the recommendations of the Brazilian National Program for Control of Blood Pressure. For analysis, individuals with systolic blood pressure (SBP) ≥ 140 mmHg and/or diastolic blood pressure (DBP) ≥ 90 mmHg were considered hypertensive.

The independent variables used in the analyses were: age in years (20 to 29; 30 to 39; 40 to 49; 50 to 59; 60 to 65; 65 to 69; 70 to 79; ≥ 80), gender (male or female), family income in minimum wages per person (< 1; 1 to 1.9; 2 to 4.9; ≥ 5), schooling in years of study (0; 1 to 4; ≥ 5), marital status (with or without a partner), smoking (smoker, former smoker, or never smoked) and sedentary lifestyle in leisure (sedentary or non-sedentary). The leisure section of the extended version of the International Physical Activity Questionnaire^18^(IPAQ) was used for this last variable. A score was created with the sum of the physical activities of low, moderate, and high intensity, classifying individuals who spent less than 150 minutes per week in them as sedentary.

The analyses were performed using the Stata 13.0 statistical package, including the calculations of proportions and their respective 95% confidence intervals (95%CI). The statistical significance of the differences between WHR means according to gender was verified by the Student’s t-test, and the analysis of variance (Anova) was used to verify the differences in the means according to age. The chi-square test was used to identify the differences between the strata of the variables studied. The statistical significance level of 5% was considered for all associations.

Poisson regression was used to calculate the unadjusted and adjusted prevalence ratios with 95%CI and significance values for heterogeneity obtained by Wald tests. The adjusted analysis verified the association between the SAH and the health risk categories, with control for potential confounding for age, schooling, family income, smoking, marital status, and leisure-time physical activity.

This study was submitted to the Ethics Committee of the Universidade Federal de Pelotas and approved under the number 152/2007.

## RESULTS

Data from 8,235 adults and 5,494 older adults were analyzed. More than 60% of the sample consisted of females, which showed higher means for BMI and WHR. Men had higher means for weight, height, WC, SBP, and DBP. Higher prevalence of overweight and changes in WC were verified in the female population, but men had higher percentages of SAH. The prevalence of SAH was 17.6% for the adult population and 23.0% for the older population. The overweight assessed by BMI, in turn, exceeded 51.0%, with higher frequencies among females (51.1% for adult women and 61.4% for older women). Regarding the overall prevalence of central overweight, 57.1% and 21.2% of adults and older adults, respectively, fulfilled this criterion, as well as 34.8% and 54.3% for central obesity; in adult women, central overweight was present in 68.1% and central obesity in 44.0%, and the percentages for the older women were 17.9% and 70.4%, respectively (Table 1).

**Table 1:** Description of the population according to gender and demographic, anthropometric, and morbidity characteristics. Brazil, 2009.

Variable	All individuals		Men		Women		p*
Adults	n = 8.235		n = 2.814		n = 5.421		
	Mean	SD	Mean	SD	Mean	SD	

Age (years)	38.1	11.48	37.8	11.78	38.2	11.32	0.192
Weight (kg)	68.45	15.18	74.80	15.11	65.15	14.13	< 0.001
Height (m)	1.63	0.093	1.71	0.073	1.58	0.068	< 0.001
BMI (kg/m^2^)	25.79	5.18	25.43	4.63	25.98	5.43	< 0.001
WC (cm)	88.19	13.27	90.42	12.89	87.03	13.81	< 0.001
WHR	0.54	0.083	0.52	0.075	0.55	0.087	< 0.001
SBP (mmHg)	123.95	19.40	128.53	18.93	121.58	19.21	< 0.001
DBP (mmHg)	81.76	13.69	83.90	14.25	80.65	13.26	< 0.001

	%	IC95%	%	IC95%	%	IC95%	

SAH (%)	17.6	16.8–18.4	22.3	20.8–23.9	15.2	14.2–16.1	< 0.001
Overweight BMI (%)	50.5	49.5–51.6	49.5	47.6–51.3	51.1	49.8–52.4	0.162
High WC (%)	57.1	56.0–58.2	35.7	34.0–37.5	68.1	66.8–69.3	< 0.001
Very high WC (%)	34.8	33.7–35.8	17.2	15.8–18.6	44.0	42.7–45.3	< 0.001

Older adults	n = 5,494		n = 2,110		n = 3,384		
	Média	DP	Média	DP	Média	DP	

Age (years)	70.9	7.99	70.9	7.75	70.9	8.16	0.850
Weight (kg)	65.3	14.15	69.3	13.73	62.48	13.84	< 0.001
Height (m)	1.57	0.093	1.65	0.074	1.53	0.068	< 0.001
BMI (kg/m^2^)	26.20	5.03	25.21	4.32	26.81	5.34	< 0.001
WC (cm)	94.57	12.44	95.33	12.26	94.11	12.53	< 0.001
WHR	0.60	0.081	0.57	0.072	0.61	0.083	< 0.001
SBP (mmHg)	137.76	23.93	138.79	24.25	137.13	23.72	0.011
DBP (mmHg)	83.79	14.53	84.70	15.02	83.22	14.19	< 0.001

	%	IC95%	%	IC95%	%	IC95%	

SAH (%)	23.3	22.3–24.5	25.6	23.8–27.5	22.0	20.6–23.4	0.002
Overweight BMI (%)	57.1	55.7–58.4	50.2	48.0–52.4	61.4	59.7–63.0	< 0.001
High WC (%)	21.2	20.1–22.2	26.5	24.6–28.4	17.9	16.7–19.2	< 0.001
Very high WC (%)	54.3	53.0–55.6	28.6	26.7–30.5	70.4	68.8–71.9	< 0.001

BMI: Body mass index; WC: waist circumference; WHR: waist-to-height ratio; SBP: systolic blood pressure; DBP: diastolic blood pressure; SAH: systolic arterial hypertension

* Student’s t-test or chi-square test for differences between men and women.

**Figure 2:**
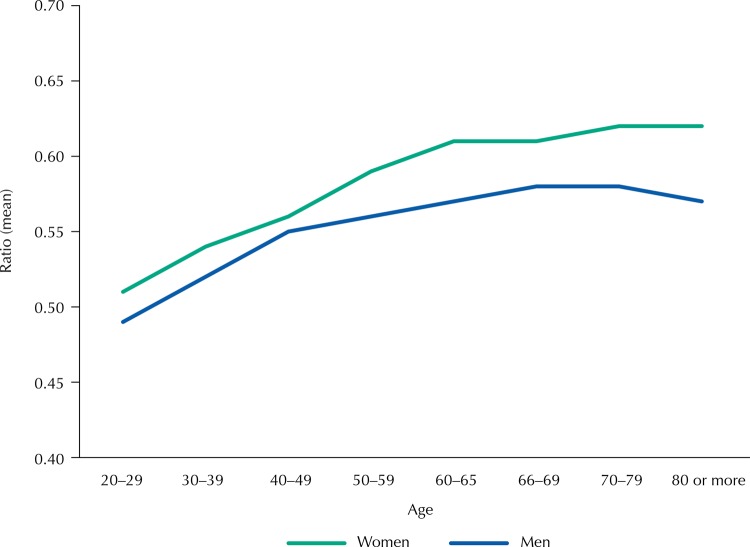
Mean values of the waist-to-height ratio according to age categories among men and women. Brazil, 2009.

WHR means according to gender and age categories are shown in Figure 2, and lower means were observed among the younger age groups. Significant differences were observed among WHR means according to the age categories for the adult population (p < 0.001), for both genders; for older, in turn, the averages did not differ significantly. The total means of WHR for adults were 0.52 (SD = 0.075) and 0.55 (SD = 0.087) for men and women, respectively, being significantly higher for the older adults: 0.57 (SD = 0.072) and 0.61 (SD = 0.083).

This study showed the cutoff point of WHR that optimizes the sensitivity/specificity ratio for the adult population, using BMI as an anthropometric reference, was 0.52 for males and 0.54 for females. The sensitivity values evaluated were 86.3% for men (95%CI 84.3–88.0) and 84.7% for women (95%CI 83.3–86.0); the percentages of 83.6 (95%CI 81.5–85.5) and 82.5 (95%CI 81.0–84.0) correspond to the specificity values for men and women, respectively, whose ROC curves showed areas of 0.84 (95%CI 0.83–0.86) and 0.83 (95%CI 0.82–0.84).

The estimates of the prevalence of overweight were calculated using WHR as an anthropometric marker for health risk (Table 2), highlighting that the cutoff point of 0.50 for all age groups and both genders encompasses the values that are also established here, as well as in a previous study^16^. A tendency of increase in the prevalence of overweight according to WHR is observed as age increases, with significant differences between genders (p < 0.001) and higher percentages among women.

**Table 2:** Distribution of the sample according to the prevalence of overweight based on the waist-to-height ratio (WHR) by age categories among men and women. Brazil, 2009.

Age (years)	Total sample	Men	Overweight (men)	Women	Overweight (men)
					
	n (%)	n (%)	% (95%CI)	n (%)	% (95%CI)
Adults			WHR ≥ 0.50		WHR ≥ 0.50

20–29	2,451 (29.8)	887 (31.5)	37.9 (34.7–41.1)	1,564 (28.8)	47.5 (45.0–50.0)
30–39	2,029 (24.6)	635 (22.6)	62.4 (58.5–66.0)	1,394 (25.7)	67.1 (64.6–69.6)
40–49	2,059 (25.0)	700 (24.9)	72.6 (69.1–75.5)	1,359 (25.1)	78.4 (76.2–80.5)
50–59	1,696 (20.6)	592 (21.0)	79.6 (76.1–82.6)	1,104 (20.4)	86.8 (84.6–88.6)
Total	8,235 (100)	2,814 (100)	60.7 (58.9–62.6)	5,421 (100)	68.2 (66.9–69.5)

Older Adults			WHR ≥ 0.50		WHR ≥ 0.50

60–65	1,372 (25.0)	489 (23.2)	87.9 (84.7–90.5)	883 (26.1)	92.3 (90.3–93.9)
65–69	1,384 (25.2)	528 (25.0)	87.1 (83.9–89.7)	856 (25.3)	93.2 (91.3–94.7)
70–79	1,950 (35.5)	790 (37.4)	87.2 (84.7–89.4)	1,160 (34.3)	93.0 (91.4–94.3)
≥ 80	788 (14.3)	303 (14.4)	84.5 (79.9–88.2)	485 (14.3)	91.9 (89.2–94.1)
Total	5,494 (100)	2,110 (100)	86,9 (85,4–88,3)	3.384 (100)	92.7 (91.7–93.5)

The prevalence of health risk by sex, based on the correlation matrix between BMI and WC for adults and older adults can be seen in Table 3. The prevalence of moderate risk ranged from 15.4% to 18.5% in the sample studied, and women showed higher percentages of high health risk, with values of 38.1% for adult women and 55.1% for older women.

**Table 3:** Distribution of adults and older adults according to health risk categories based on the correlation matrix between the body mass index (BMI) and the waist circumference (WC) and the waist-to-height ratio (WHR). Brazil, 2009.

Health risk categories (BMI and WC)	Men		Women	
	
	n (%)	n (%)	n (%)	n (%)
Adults	BMI + WC	WHR ≥ 0.50	BMI + WC	WHR ≥ 0.50

Low risk	1,786 (64.9)	707 (39.6)	2,459 (46.5)	819 (33.3)
Moderate risk	510 (18.5)	508 (99.6)	814 (15.4)	773 (95.0)
High risk	457 (16.6)	457 (100)	2,013 (38.1)	2,013 (100)

Older adults		RCE ≥ 0.50		RCE ≥ 0,50

Low risk	1,038 (52.6)	849 (81.8)	702 (22.3)	555 (79.1)
Moderate risk	368 (18.7)	368 (100)	585 (18.5)	584 (99.8)
High risk	566 (28.7)	566 (100)	1,868 (59.2)	1,868 (100)

The analyses of the health risk categories using WHR as an anthropometric marker (Table 3) allowed us to observe that, in the sample classified as low risk using the correlation matrix between BMI and WC, 39.6% of adult men, 33.3% of adult women and more than 79% of the older of both genders showed a WHR showing increased risk. However, only 5% of adult women and 0.2% of the older adults in the group classified as moderate risk by the correlation matrix between BMI and WC were diagnosed as low risk by WHR.

The unadjusted analysis showed the prevalence ratio for SAH was about three times higher for adult individuals classified in the high health risk category using the correlation matrix between BMI and WC, compared with the reference category (low risk). All health risk categories continued to show association with SAH after adjustment for potential confounding factors, and prevalence ratios remained almost two times higher than the reference value for individuals classified at high risk. Prevalence ratios very close to the correlation matrix between BMI and WC were observed when WHR was used as a health risk marker, both in unadjusted and adjusted analyses (Table 4).

**Table 4:** Unadjusted and adjusted prevalence ratio (PR) for hypertension in adults and older adults according to health risk categories by the combination matrix between BMI and WC and the waist-to-height ratio among men and women. Brazil, 2009.

Variable	Men		Women	
	
	Unadjusted PR (95%CI)	Adjusted PR* (95%CI)	Unadjusted PR (95%CI)	Adjusted PR* (95%CI)
Adults				

Combination Matrix (BMI and WC)				
Low risk	1.00	1.00	1.00	1.00
Moderate risk	1.87 (1.53–2.28)	1.68 (1.36–2.07)	1.68 (1.34–2.11)	1.35 (1.07–1.71)
High risk	2.81 (2.35–3.37)	2.42 (1.99–2.94)	2.98 (2.55–3.49)	2.11 (1.79–2.50)
Waist-to-height ratio (WHR)				
Low risk	1.00	1.00	1.00	1.00
Increased risk	2.44 (2.04–2.92)	2.01 (1.67–2.42)	2.86 (2.36–3.46)	1.70 (1.39–2.08)

Older adults				

Combination Matrix (BMI and WC)				
Low risk	1.00	1.00	1.00	1.00
Moderate risk	1.29 (1.05–1.57)	1.26 (1.03–1.54)	1.34 (1.10–1.62)	1.32 (1.08–1.60)
High risk	1.57 (1.34–1.84)	1.56 (1.33–1.84)	1.34 (1.15–1.57)	1.29 (1.10–1.51)
Waist-to-height ratio (WHR)				
Low risk	1.00	1.00	1.00	1.00
Increased risk	1.70 (1.28–2.25)	1.71 (1.28–2.29)	1.63 (1.19–2.26)	1.53 (1.10–2.12)

BMI: body mass index; WC: waist circumference

* Adjustment for age, schooling, income, smoking, marital status, and leisure-time physical activity.

An increase of 26% was observed (PR = 1.26; 95%CI 1.03–1.54) in the prevalence of SAH for older adults in the moderate health risk category (Table 4); in the high-risk category, this increase was of 56% (PR = 1.56; 95%CI 1.33–1.84) in relation to the category of individuals without risk. However, the prevalence for older women increased, with percentages of 32.0% (PR = 1.32; 95%CI 1.08–1.60) and 29.0% (PR = 1.29; 95%CI 1.10–1.51) for the categories moderate and high risk, respectively. Higher values in the prevalence ratios of SAH both for men (PR = 1.71; 95%CI 1.28–2.29) and for women (PR = 1.53; 95%CI 1.10–2.12) were observed when using WHR as a health risk marker.

## DISCUSSION

The search for a simple anthropometric predictor showing previous ability to identify complications and chronic non-communicable diseases has been increasing worldwide. This study highlights WHR and the combination matrix between BMI and WC have comparable abilities to identify individuals with SAH, regardless of the latter’s gender and age. In fact, WHR was able to identify SAH using the cutoff point of 0.50 as a reference for health risk in a representative sample. These results are unprecedented in Brazil.

For this reason and as it is an effective, practical and easy-to-interpret cardiometabolic risk marker, we could do no other than propagandize the routine use of WHR as an anthropometric health risk marker, both in epidemiological studies and in the individual and collective clinical practice. The substitution of the correlation matrix between BMI and WC for WHR is the most relevant element in the discussion presented here.

Regarding the cutoff points of WHR as an anthropometric marker for the adult population, the results agree with the international literature^19^. In the sample analyzed, using the values of 0.52 and 0.54 allows identifying a large portion of overweight individuals, since it must correctly classify from 84% to 88% of men and from 83% to 86% of women, which shows it is a valuable anthropometric marker for diagnosis of this nutritional disorder.

WHR is more advantageous than WC because it presupposes that, for a certain height, a specific amount of trunk fat is acceptable, thus allowing setting a single cutoff point applicable to the general population, regardless of gender and age^12^. Studies aiming to identify cutoff points of WHR and to compare them with other anthropometric measurements of overweight or discriminators of cardiometabolic risk factors have found values higher than 0.50 indicating health risk^20,21^.

In Brazil, a study conducted with a specific population of adults and older adults participating in the program for monitoring cardiovascular diseases and diabetes determined the cutoff points of 0.52 for WHR for men and of 0.53 for women^22^. A study conducted by Rodrigues et al.^23^, aimed to test the association between WHR and cardiovascular risk factors, observed the cutoff points for WHR of 0.52 and 0.53 for hypertension and 0.53 and 0.54 for metabolic syndrome, for men and women, respectively. They concluded that WHR was more efficient than other anthropometric measurements in the ability to identify such risk factors.

It is already well documented in the literature worldwide that gender and age are risk factors for overweight, regardless of the anthropometric marker used^2,24^. These findings agree with those observed in this study, in which a tendency of increase in the median values of WHR is observed with increasing age, with significant differences in function of gender. According to recent studies, the continuous progression of fat accumulation evaluated using WHR represents a possible increase in the cardiometabolic risk^25^.

Similarly to national surveys^26,27^, the study in question found a higher frequency of overweight among women. Using WHR as an assessment tool, prevalence ranging from 60% to 68% for adults and from 86% to 92% for the older adults reinforce the evidence that overweight is one of the major problems in public health^28^.

Although BMI is an internationally accepted method for classification of nutritional status, its adoption as a single classification pattern can result in inaccurate assessments and, consequently, in erroneous diagnoses, leading to possible interventions inappropriate to the treatment for overweight^5^. The assumption that BMI measurements adiposity in all age groups and with the same ability may be mistaken^29^. The anthropometric measurements evaluating abdominal fat accumulation, such as WHR, have shown a higher predictive ability for chronic non-communicable diseases; therefore, they are recommended for evaluation of the individuals’ health, regardless of their body weight^12^.

Studies have proven that people with normal weight or whose overweight was diagnosed by BMI may have a higher number of underestimated morbidities when their WC increases simultaneously^6,10^. In Brazil, Meller et al.^30^ conducted a study with adult women and found that one out of four women without overweight had a WC > 80 cm. Similar results were observed in a study conducted in Maranhão^31^, which found 15.5% of abdominal obesity in eutrophic women. Thus, for a more accurate assessment of health risk in individuals or populations, several researchers have recommended the combined use of BMI and WC^6^ to increase the accuracy in the diagnosis of this nutritional disorder, which predicts countless diseases and health problems.

Even recognizing that the combination of anthropometric measurements can increase the sensitivity in the identification of health risk, few studies conducted in the country^31^ investigated individuals showing change in WC simultaneously with overweight. The data from this study show important differences in the risk diagnosis between genders, with higher prevalence for both adult and older women. Studies by Veloso and Silva^31^and Soares and Barreto^33^corroborate these results.

International studies point that the combination of BMI and WC increases the probability of detecting chronic non-communicable diseases, namely hypertension, diabetes, and dyslipidemia, in relation to the isolated use of these measurements^34^. Considering that, we must question the practicality of combining these measurements in the clinical practice of health professionals. The search for a simple marker that can efficiently screen a greater number of individuals in programs for health promotion and prevention of non-communicable diseases and complications has played a central role in health risk discussions. Therefore, Ashwell and Gibson^11^ recommend substituting the association between BMI and WC with the routine use of WHR, arguing that it is a simple, easy-to-interpret and low-cost primary risk assessment tool, which identifies a higher number of people at cardiometabolic risk.

In this study, 39.6% and 33.3% of adult men and women and 81.8% and 79.1% of older men and women, respectively, categorized as low risk by the combination between BMI and WC showed increased risk when classified by WHR; therefore, they were at risk of not being warned about the need for actions for health promotion and prevention of non-communicable diseases and complications, which is similar to the findings by Ashwell and Gibson^11^. A study conducted with 36,642 adult Thai individuals also corroborates the results of this study, showing WHR was capable of identifying more individuals at cardiometabolic risk, even if they were categorized as “healthy” or “normal” according to BMI or WC^36^.

The international^37^ and Brazilian^38^ literatures confirm a high explanatory power for both BMI and WC in the prevalence of SAH, and such power is increased when combining the two measurements^34^. In this study, the ability of WHR to determine prevalence ratios for SAH was similar to that of the association between the BMI and WC anthropometric indicators, considering that there was overlapping of the confidence intervals for the increased risk assessed by WHR with the moderate and high risks assessed by the combination matrix, both in adults and in older adults.

In sum, the results of this study confirm recent data from the literature, which show the high discriminatory power of WHR in the early identification of individuals at health risk, besides having abilities similar to that of the measurements of adiposity combined to identify prevalence ratios of SAH. Thus, WHR proves to be an important health risk marker, which is similar to adiposity measurements, regardless of aging.

Finally, we encourage the inclusion of WHR in the routine of services and in the planning of health actions, as well as in epidemiological studies. Health education through the message “keep your waist circumference to less than half your height” will be more understandable and effective, in all age groups, as an attribute of a healthy life.
