# Supplementary material for: The ability of waist-to-height ratio to identify health risk
Source: Rev Saude Publica. 2019 Sep 12;53:66. doi: 10.11606/s1518-8787.2019053000895 (PMC6752642; doi:10.11606/s1518-8787.2019053000895)
Supplement: Translation

# Translation: Habilidade da razão cintura-estatura na identificação de risco à saúde

**Authors:** Márcia Mara Corrêa, Luiz Augusto Facchini, Elaine Thumé, Elizabete Regina Araújo de Oliveira, Elaine Tomasi

**Affiliations:** I Universidade Federal de Pelotas. Programa de Pós-Graduação em Epidemiologia. Pelotas, RS, Brasil; II Universidade Federal de Pelotas. Programa de Pós-Graduação em Enfermagem. Pelotas, RS, Brasil; III Universidade Federal do Espírito Santo. Programa de Pós-Graduação em Saúde Coletiva. Vitória, ES, Brasil

**Keywords:** Índice de Massa Corporal, Circunferência da Cintura, Estatura, Sobrepeso, Fatores de Risco, Hipertensão, diagnóstico

## Abstract

**OBJETIVO:**

Avaliar o desempenho da razão entre a circunferência da cintura e a estatura na identificação de risco à saúde comparada à matriz de associação entre os parâmetros antropométricos índice de massa corporal e circunferência da cintura.

**MÉTODOS:**

Estudo de base populacional apresentando um corte transversal em uma amostra representativa da população adulta e idosa brasileira. A combinação do índice de massa corporal com a circunferência da cintura deu origem às categorias de risco à saúde, assim como os pontos de corte da razão entre a circunferência da cintura e a estatura como indicador antropométrico foram utilizados para a classificação de baixo risco e risco aumentado. A regressão de Poisson foi utilizada para verificar a associação da hipertensão arterial sistêmica com as categorias de risco à saúde.

**RESULTADOS:**

Os resultados apontaram que 26% dos homens adultos, 10,4% das mulheres adultas e mais de 30% dos idosos de ambos os sexos classificados como sem risco pela matriz de combinação índice de massa corporal e circunferência da cintura apresentaram razão entre a circunferência da cintura e a estatura indicativa de risco aumentado. Todas as categorias de risco continuaram apresentando associação com a hipertensão após controle para os fatores de confusão, permanecendo próximas de duas vezes maiores para os indivíduos adultos com risco moderado e elevado segundo ambos os métodos. Já os idosos apresentaram razões de prevalência de hipertensão de 1,37 (IC95% 1,16–1,63) e de 1,35 (IC95% 1,12–1,62), para homens e mulheres, respectivamente, quando utilizada a razão entre a circunferência da cintura e a estatura como indicador de risco, estando estes valores próximos à matriz de combinação índice de massa corporal e circunferência da cintura.

**CONCLUSÕES:**

A razão entre a circunferência da cintura e a estatura identificou mais indivíduos em risco precoce à saúde do que a matriz de combinação entre o índice de massa corporal e a circunferência da cintura e apresentou habilidades comparáveis na identificação de risco à saúde, independentemente do sexo e da faixa etária, no que tange às razões de prevalência para hipertensão arterial sistêmica.

## INTRODUÇÃO

A obesidade e, mais recentemente, o sobrepeso têm sido reconhecidos como grandes problemas de saúde pública em muitos países^1^, incluindo o Brasil^2^, e várias tentativas têm sido realizadas para identificar o melhor preditor antropométrico^3^ para diversas doenças e agravos não transmissíveis em diferentes populações e faixas etárias. Para o diagnóstico do excesso de peso, várias técnicas têm sido propostas para estimar com precisão a quantidade total de gordura corporal, assim como sua distribuição^4^. Considerando-se a facilidade metodológica e os custos desses métodos, tanto na realização de estudos epidemiológicos como na prática clínica, tem-se preconizado o uso do índice de massa corporal (IMC)^1^ e da circunferência da cintura (CC)^1^ como indicadores antropométricos eficazes neste tipo de avaliação.

Como a relação entre o IMC e o risco de morbidades pode ser afetada pela distribuição da gordura corpórea, independentemente do peso corporal^5^, estudos têm recomendado que esse índice seja combinado a outras medidas de adiposidade abdominal, com destaque para a CC, para um melhor diagnóstico do excesso de peso como preditor de risco à saúde^6^. O excesso de gordura abdominal tem sido associado a distúrbios no metabolismo de glicose e lipídios, os quais se relacionam com as doenças cardiovasculares, resistência à insulina e hipertensão arterial sistêmica (HAS)^9^. Por esses motivos, tem-se recomendado a CC como marcador antropométrico de risco cardiometabólico^6,10^.

Entretanto, estudo recente sugere o uso da razão entre a circunferência da cintura e a estatura (RCE) como medida antropométrica substituta à matriz de associação IMC e CC por sua maior capacidade de identificar indivíduos em risco à saúde, além de estar fortemente associada aos fatores de risco cardiovasculares e metabólicos, independentemente do peso corporal^11^. Revisão sistemática e metanálise realizada em 2012 com mais de 300.000 indivíduos concluiu que a RCE é a melhor ferramenta de triagem para detectar fatores de risco cardiometabólicos em ambos os sexos e diversos grupos étnicos, evidenciando sua superioridade sobre o IMC e a CC^12^.

A utilização em estudos epidemiológicos e na prática clínica, tanto individual como coletiva, de uma medida simples, de fácil interpretação e de baixo custo – e principalmente que possa ser realizada como forma de triagem em programas de promoção da saúde e prevenção de doenças e agravos não transmissíveis – incitou a realização deste estudo. O objetivo foi avaliar a habilidade da RCE na identificação de risco à saúde, com destaque para a HAS, comparada à matriz de associação entre os índices antropométricos IMC e CC. No Brasil, ainda não houve estudo de base populacional que utilizasse a RCE como indicador antropométrico de risco à saúde em amostra representativa da população adulta e idosa.

## MÉTODOS

Este estudo é parte de um inquérito epidemiológico de base populacional, realizado em 2008 e 2009, que teve como objetivo avaliar o acesso e a qualidade da atenção da rede de saúde em residentes das áreas urbanas de 100 municípios dos 23 estados brasileiros. Trata-se de um estudo de delineamento transversal, de base populacional, em amostra representativa da população adulta e idosa brasileira, sendo considerados inelegíveis para o estudo os indivíduos hospitalizados, privados de liberdade por decisão judicial, ou residindo em instituições de longa permanência. O inquérito foi composto por uma amostra total de 13.756 adultos e 7.015 idosos. O percentual de perdas e recusas foi, respectivamente, de 8% e 2% para a população adulta e de 4% e 2% para a idosa. As entrevistas realizadas com informantes-chave, nas quais não havia dados antropométricos (3.998 adultos e 1.128 idosos), foram excluídas, obtendo-se assim uma amostra final de 8.235 adultos e 5.494 idosos com as medidas antropométricas necessárias para as análises. Essa amostra apresentou poder estatístico superior a 95% para as associações testadas.

Para a seleção de municípios e setores censitários urbanos, foram utilizados os dados do Censo Populacional Brasileiro de 2000, realizado pelo Instituto Brasileiro de Geografia e Estatística (IBGE)^13^. O padrão de referência territorial e populacional para as estimativas amostrais foi o setor censitário urbano, definido como um agregado de aproximadamente 300 domicílios e 1.000 habitantes, sendo os municípios agrupados por porte populacional, assim denominados: “muito pequenos” os com menos de 10 mil habitantes, “pequenos” os de 10 mil até 20 mil habitantes, “médios” os de 20 mil até 100 mil habitantes, “grandes” os de 100 mil até 1,1 milhão de habitantes, e “muito grandes” aqueles a partir de 1,1 milhão de habitantes. Em cada município, os setores censitários foram sorteados aleatoriamente, e em cada um deles procedeu-se à identificação de amostras independentes de adultos e idosos. Para as amostras de adultos, eram visitados 10 domicílios, enquanto para as de idosos, 30 domicílios, seguindo um “salto” sistemático entre as residências. Com essa estratégia, esperava-se entrevistar aproximadamente 19 adultos e 10 idosos por setor. Em cada domicílio foram incluídos todos os indivíduos elegíveis, ainda que se ultrapassasse a cota pré-definida.

A coleta de dados foi realizada por auxiliares de pesquisa devidamente treinados, sendo os questionários estruturados em cinco blocos: identificação, promoção da saúde e cuidados preventivos, problemas de saúde, acesso e utilização de serviços de saúde e medidas antropométricas. Os questionários estavam disponíveis em computador do tipo *palmtop* (*personal digital assistant* ou PDA).

Utilizando as técnicas propostas por Lohman et al.^14^, as variáveis antropométricas peso, altura e CC foram aferidas duas vezes, sendo o resultado final de cada variável obtido por meio do cálculo da média aritmética. A CC foi obtida entre a crista ilíaca e o rebordo costal lateral (ponto médio entre o quadril e a última costela) com precisão de 0,1 cm.

Para o diagnóstico de acúmulo de gordura na região abdominal, foram considerados os valores da CC, sendo classificados como baixo acúmulo os valores menores que 80 e 94 cm para mulheres e homens, respectivamente. Valores entre 94 e 102 cm para homens e entre 80 e 88 cm para mulheres foram considerados como alto acúmulo; acima de 102 cm para homens e de 88 cm para mulheres, como muito alto^1^.

A partir da divisão do peso corporal (kg) pela altura (m) elevada ao quadrado (P/A^2^), foi calculado o IMC, cuja classificação foi realizada com base na referência proposta pela Organização Mundial da Saúde (OMS)^1^. Segundo Molarius et al.^15^ a associação da medida do IMC com a CC oferece uma forma combinada de avaliação de risco à saúde, além de diminuir as limitações de cada uma das medidas de forma isolada. A matriz de associação dessas medidas originou as categorias de risco à saúde, conforme demonstrado na Figura 1.

**Figura 1:** Categorias de risco à saúde utilizando a matriz de associação entre o IMC e a circunferência da cintura. IMC: índice de massa corporal

A RCE foi calculada utilizando a medida da CC dividida pela altura, ambas em centímetros (cm), sendo o resultado máximo da equação igual a um. No Brasil, estudo recente de base populacional estabeleceu o valor de 0,55 como ponto de corte da RCE como indicador antropométrico de excesso de peso em indivíduos idosos^16^, utilizando o IMC como referência antropométrica. No entanto, não foi encontrado na literatura nacional um valor de referência do ponto de corte da RCE como marcador de excesso de peso para os indivíduos adultos; portanto, ele foi estabelecido utilizando a curva ROC (*receiver operating characteristic*, ou característica de operação do receptor). A área sob a curva ROC (AUROC) e pontos de corte da RCE com maiores valores de sensibilidade e especificidade foram utilizados como critérios para identificar excesso de peso, utilizando o IMC como referência antropométrica. O ponto de corte 0,50, tanto para adultos como idosos, foi utilizado para classificação entre baixo risco e risco aumentado como categorias de risco à saúde.

A pressão arterial (PA) foi aferida utilizando aparelho digital de pulso automático, sendo realizadas duas medidas com intervalo mínimo de quinze minutos entre elas, seguindo as recomendações do Programa Nacional de Controle de Hipertensão Arterial^17^. Para fins de análise, considerou-se como hipertensos os indivíduos com pressão arterial sistólica (PAS) ≥ 140 mmHg e/ou pressão arterial diastólica (PAD) ≥ 90 mmHg.

As variáveis independentes utilizadas nas análises foram: idade em anos (20 a 29; 30 a 39; 40 a 49; 50 a 59; 60 a 65; 65 a 69; 70 a 79; ≥ 80), sexo (masculino ou feminino), renda familiar em salários mínimos per capita (< 1; 1 a 1,9; 2 a 4,9; ≥ 5), escolaridade em anos de estudo (0; 1 a 4; ≥ 5), situação conjugal (com ou sem companheiro), tabagismo (fumante, ex-fumante ou nunca fumou) e sedentarismo no lazer (sedentário ou não sedentário). A seção de lazer da versão longa do *International Physical Activity Questionnaire*^18^ (IPAQ) foi utilizada para essa última variável. Foi construído um escore com a soma das atividades físicas de baixa, moderada e alta intensidade, sendo classificados como sedentários os indivíduos que despendiam menos de 150 minutos por semana nelas.

As análises foram realizadas utilizando o pacote estatístico Stata 13.0, sendo incluídos os cálculos das proporções e seus respectivos intervalos de 95% de confiança (IC95%). A significância estatística das diferenças entre as médias de RCE conforme o sexo foi verificada por meio do teste t de Student, e a análise de variância (Anova) foi utilizada para verificar as diferenças nas médias de acordo com a idade. Utilizou-se o teste qui-quadrado para identificar as diferenças entre os estratos das variáveis estudadas. O nível de significância estatística de 5% foi considerado para todas as associações.

A regressão de Poisson foi utilizada para os cálculos das razões de prevalência não ajustadas e ajustadas com IC95% e valores de significância obtidos pelos testes de Wald para heterogeneidade. A análise ajustada verificou a associação entre HAS e as categorias de risco à saúde, com controle de potencial confusão para idade, escolaridade, renda familiar, tabagismo, situação conjugal e atividade física no lazer.

O estudo foi submetido ao Comitê de Ética da Universidade Federal de Pelotas e aprovado sob o número 152/2007.

## RESULTADOS

Foram analisados os dados de 8.235 adultos e 5.494 idosos. Mais de 60% da amostra foi composta pelo sexo feminino, que apresentou maiores médias de IMC e RCE. Os homens apresentaram médias mais altas de peso, altura, CC, PAS e PAD. Prevalências mais elevadas de excesso de peso e alterações na CC foram verificadas na população feminina, mas os homens apresentaram percentuais mais elevados de HAS. As prevalências de HAS foram de 17,6% para a população adulta e de 23,0% para a idosa. Já o excesso de peso avaliado pelo IMC ultrapassou os 51,0%, apresentando maiores frequências no sexo feminino (51,1% para as adultas e 61,4% para idosas). Quanto à prevalência geral de sobrepeso central, 57,1% e 21,2% dos adultos e idosos, respectivamente, preenchiam esse critério, assim como 34,8% e 54,3% para obesidade central; nas mulheres adultas, o sobrepeso central esteve presente em 68,1% e a obesidade central em 44,0%, enquanto os percentuais nas idosas foram de 17,9% e 70,4%, respectivamente (Tabela 1).

**Tabela 1:** Descrição da população por sexo e segundo características demográficas, antropométricas e de morbidade. Brasil, 2009.

Variável	Todos os indivíduos		Homens		Mulheres		p*
Adultos	n = 8.235		n = 2.814		n = 5.421		
	Média	DP	Média	DP	Média	DP	

Idade (anos)	38,1	11,48	37,8	11,78	38,2	11,32	0,192
Peso (kg)	68,45	15,18	74,80	15,11	65,15	14,13	< 0,001
Altura (m)	1,63	0,093	1,71	0,073	1,58	0,068	< 0,001
IMC (kg/m^2^)	25,79	5,18	25,43	4,63	25,98	5,43	< 0,001
CC (cm)	88,19	13,27	90,42	12,89	87,03	13,81	< 0,001
RCE	0,54	0,083	0,52	0,075	0,55	0,087	< 0,001
PAS (mmHg)	123,95	19,40	128,53	18,93	121,58	19,21	< 0,001
PAD (mmHg)	81,76	13,69	83,90	14,25	80,65	13,26	< 0,001
	%	IC95%	%	IC95%	%	IC95%	
HAS (%)	17,6	16,8–18,4	22,3	20,8–23,9	15,2	14,2–16,1	< 0,001
Excesso de peso IMC (%)	50,5	49,5–51,6	49,5	47,6–51,3	51,1	49,8–52,4	0,162
CC alta (%)	57,1	56,0–58,2	35,7	34,0–37,5	68,1	66,8–69,3	< 0,001
CC muito alta (%)	34,8	33,7–35,8	17,2	15,8–18,6	44,0	42,7–45,3	< 0,001

Idosos	n = 5.494		n = 2.110		n = 3.384		
	Média	DP	Média	DP	Média	DP	

Idade (anos)	70,9	7,99	70,9	7,75	70,9	8,16	0,850
Peso (kg)	65,3	14,15	69,3	13,73	62,48	13,84	< 0,001
Altura (m)	1,57	0,093	1,65	0,074	1,53	0,068	< 0,001
IMC (kg/m^2^)	26,20	5,03	25,21	4,32	26,81	5,34	< 0,001
CC (cm)	94,57	12,44	95,33	12,26	94,11	12,53	< 0,001
RCE	0,60	0,081	0,57	0,072	0,61	0,083	< 0,001
PAS (mmHg)	137,76	23,93	138,79	24,25	137,13	23,72	0,011
PAD (mmHg)	83,79	14,53	84,70	15,02	83,22	14,19	< 0,001

	%	IC95%	%	IC95%	%	IC95%	

HAS (%)	23,3	22,3–24,5	25,6	23,8–27,5	22,0	20,6–23,4	0,002
Excesso de peso IMC (%)	57,1	55,7–58,4	50,2	48,0–52,4	61,4	59,7–63,0	< 0,001
CC alta (%)	21,2	20,1–22,2	26,5	24,6–28,4	17,9	16,7–19,2	< 0,001
CC muito alta (%)	54,3	53,0–55,6	28,6	26,7–30,5	70,4	68,8–71,9	< 0,001

IMC: índice de massa corporal; CC: circunferência da cintura; RCE: razão cintura-estatura; PAS: pressão arterial sistólica; PAD: pressão arterial diastólica; HAS: hipertensão arterial

* teste t de Student ou teste qui-quadrado para as diferenças entre homens e mulheres.

As médias da RCE por gênero e segundo categorias de idade estão representadas na Figura 2, sendo possível observar médias menores entre as faixas etárias mais jovens. Observaram-se diferenças importantes entre as médias da RCE segundo as categorias de idade para a população adulta (p < 0,001), para ambos os sexos; já entre os idosos as médias não variaram significativamente. As médias totais da RCE para os adultos foram de 0,52 (DP = 0,075) e de 0,55 (DP = 0,087) para homens e mulheres, respectivamente, sendo significativamente maiores para os idosos: 0,57 (DP = 0,072) e 0,61 (DP = 0,083).

**Figura 2:** Médias da razão cintura-estatura por categorias de idade entre homens e mulheres. Brasil, 2009.

O presente estudo evidenciou que o ponto de corte da RCE que otimiza a relação sensibilidade/especificidade para a população adulta, utilizando o IMC como referência antropométrica, foi de 0,52 para o sexo masculino e de 0,54 para o feminino. Os valores de sensibilidade avaliados foram de 86,3% para os homens (IC95% 84,3–88,0) e de 84,7% para as mulheres (IC95% 83,3–86,0); já os percentuais de 83,6 (IC95% 81,5–85,5) e de 82,5 (IC95% 81,0–84,0) correspondem aos valores de especificidade para homens e mulheres, respectivamente, cujas curvas ROC apresentaram áreas de 0,84 (IC95% 0,83–0,86) e de 0,83 (IC95% 0,82–0,84).

As estimativas das prevalências de excesso de peso utilizando a RCE como marcador antropométrico de risco à saúde foram calculadas (Tabela 2), destacando-se que o ponto de corte de 0,50 para todas as faixas etárias e ambos os sexos contempla os valores também aqui estabelecidos, assim como em estudo prévio^16^. Observa-se uma tendência de aumento nas prevalências de excesso de peso segundo a RCE com o avançar da idade, com diferenças significativas entre os gêneros (p < 0,001), constatando-se maiores percentuais entre as mulheres.

**Tabela 2:** Distribuição da amostra segundo prevalência de excesso de peso com base na razão cintura-estatura (RCE) por categorias de idade entre homens e mulheres. Brasil, 2009.

Idade (anos)	Amostra total	Homens	Excesso de peso (homens)	Mulheres	Excesso de peso (mulheres)
				
	n (%)	n (%)	% (IC95%)	n (%)	% (IC95%)
Adultos			RCE ≥ 0,50		RCE ≥ 0,50

20–29	2.451 (29,8)	887 (31,5)	37,9 (34,7–41,1)	1.564 (28,8)	47,5 (45,0–50,0)
30–39	2.029 (24,6)	635 (22,6)	62,4 (58,5–66,0)	1.394 (25,7)	67,1 (64,6–69,6)
40–49	2.059 (25,0)	700 (24,9)	72,6 (69,1–75,5)	1.359 (25,1)	78,4 (76,2–80,5)
50–59	1.696 (20,6)	592 (21,0)	79,6 (76,1–82,6)	1.104 (20,4)	86,8 (84,6–88,6)
Total	8.235 (100)	2.814 (100)	60,7 (58,9–62,6)	5.421 (100)	68,2 (66,9–69,5)

Idosos			RCE ≥ 0,50		RCE ≥ 0,50

60–65	1.372 (25,0)	489 (23,2)	87,9 (84,7–90,5)	883 (26,1)	92,3 (90,3–93,9)
65–69	1.384 (25,2)	528 (25,0)	87,1 (83,9–89,7)	856 (25,3)	93,2 (91,3–94,7)
70–79	1.950 (35,5)	790 (37,4)	87,2 (84,7–89,4)	1.160 (34,3)	93,0 (91,4–94,3)
≥ 80	788 (14,3)	303 (14,4)	84,5 (79,9–88,2)	485 (14,3)	91,9 (89,2–94,1)
Total	5.494 (100)	2.110 (100)	86,9 (85,4–88,3)	3.384 (100)	92,7 (91,7–93,5)

As prevalências de risco à saúde por sexo, baseadas na matriz de associação entre o IMC e CC, para adultos e idosos, podem ser visualizadas na Tabela 3. As prevalências de risco moderado variaram entre 15,4% e 18,5% na amostra estudada, e as mulheres apresentaram percentuais mais altos de risco elevado à saúde, com valores de 38,1% para as adultas e 55,1% para as idosas.

**Tabela 3:** Distribuição de adultos e idosos segundo categorias de risco à saúde baseadas na matriz de associação entre o índice de massa corporal (IMC) e circunferência da cintura (CC) e na razão cintura-estatura (RCE). Brasil, 2009.

Categorias de risco à saúde (IMC e CC)	Homens		Mulheres	
	
	n (%)	n (%)	n (%)	n (%)
Adultos	IMC + CC	RCE ≥ 0,50	IMC + CC	RCE ≥ 0,50

Baixo risco	1.786 (64,9)	707 (39,6)	2.459 (46,5)	819 (33,3)
Risco moderado	510 (18,5)	508 (99,6)	814 (15,4)	773 (95,0)
Risco elevado	457 (16,6)	457 (100)	2.013 (38,1)	2.013 (100)

Idosos		RCE ≥ 0,50		RCE ≥ 0,50

Baixo risco	1.038 (52,6)	849 (81,8)	702 (22,3)	555 (79,1)
Risco moderado	368 (18,7)	368 (100)	585 (18,5)	584 (99,8)
Risco elevado	566 (28,7)	566 (100)	1.868 (59,2)	1.868 (100)

As análises das categorias de risco à saúde utilizando a RCE como marcador antropométrico (Tabela 3) permitiram verificar que, na amostra classificada como de baixo risco pela matriz de associação entre o IMC e a CC, 39,6% dos homens adultos, 33,3% das mulheres adultas e mais de 79% dos idosos de ambos os sexos apresentaram RCE indicativa de risco aumentado. No entanto, apenas 5% das mulheres adultas e 0,2% das idosas do grupo classificado como risco moderado pela matriz de associação IMC e CC foram diagnosticadas como baixo risco pela RCE.

A análise não ajustada mostrou que a razão de prevalência para a HAS foi cerca de três vezes maior para os indivíduos adultos classificados na categoria de risco à saúde elevado utilizando a matriz de associação IMC e CC, comparado com a categoria de referência (baixo risco). Todas as categorias de risco à saúde continuaram apresentando associação com a HAS após ajuste para potenciais fatores de confusão, sendo que as razões de prevalência permaneceram próximas de duas vezes maiores que o valor de referência para os indivíduos classificados em risco elevado. Razões de prevalência bem próximas à matriz de associação do IMC e CC foram constatadas quando se utilizou a RCE como marcador de risco à saúde, tanto nas análises brutas como nas ajustadas (Tabela 4).

**Tabela 4:** Razão de prevalências (RP) bruta e ajustada para hipertensão em adultos e idosos segundo categorias de risco à saúde pela matriz de combinação de IMC e CC e pela razão cintura-estatura entre homens e mulheres. Brasil, 2009.

Variável	Homens		Mulheres	
	
	RP bruta (IC95%)	RP ajustada* (IC95%)	RP bruta (IC95%)	RP ajustada* (IC95%)
Adultos				

Matriz de combinação (IMC e CC)				
Baixo risco	1,00	1,00	1,00	1,00
Risco moderado	1,87 (1,53–2,28)	1,68 (1,36–2,07)	1,68 (1,34–2,11)	1,35 (1,07–1,71)
Risco elevado	2,81 (2,35–3,37)	2,42 (1,99–2,94)	2,98 (2,55–3,49)	2,11 (1,79–2,50)
Razão cintura-estatura (RCE)				
Baixo risco	1,00	1,00	1,00	1,00
Risco aumentado	2,44 (2,04–2,92)	2,01 (1,67–2,42)	2,86 (2,36–3,46)	1,70 (1,39–2,08)

Idosos				

Matriz de combinação (IMC e CC)				
Baixo risco	1,00	1,00	1,00	1,00
Risco moderado	1,29 (1,05–1,57)	1,26 (1,03–1,54)	1,34 (1,10–1,62)	1,32 (1,08–1,60)
Risco elevado	1,57 (1,34–1,84)	1,56 (1,33–1,84)	1,34 (1,15–1,57)	1,29 (1,10–1,51)
Razão cintura-estatura (RCE)				
Baixo risco	1,00	1,00	1,00	1,00
Risco aumentado	1,70 (1,28–2,25)	1,71 (1,28–2,29)	1,63 (1,19–2,26)	1,53 (1,10–2,12)

IMC: índice de massa corporal; CC: circunferência da cintura

* Ajuste para idade, escolaridade, renda, tabagismo, situação conjugal e atividade física no lazer.

Observou-se um aumento de 26% (RP = 1,26; IC95% 1,03–1,54) nas prevalências de HAS para os indivíduos idosos categorizados em risco moderado à saúde (Tabela 4); na categoria de risco elevado, esse incremento foi de 56% (RP = 1,56; IC95% 1,33–1,84) em relação à categoria dos indivíduos com ausência de risco. No entanto, as idosas apresentaram prevalências aumentadas, com percentuais de 32,0% (RP = 1,32; IC95% 1,08–1,60) e de 29,0% (RP = 1,29; IC95% 1,10–1,51) para as categorias de risco moderado e elevado, respectivamente. Valores mais elevados nas razões de prevalência de HAS tanto para homens (RP = 1,71; IC95% 1,28–2,29) como para mulheres (RP = 1,53; IC95% 1,10–2,12) foram observados quando foi empregada a RCE como indicador de risco à saúde.

## DISCUSSÃO

A procura por um preditor antropométrico simples que apresente habilidade prévia na identificação de agravos e doenças crônicas não transmissíveis vem aumentando em todo o mundo. O presente estudo destaca que a RCE e a matriz de combinação entre o IMC e CC apresentam habilidades comparáveis na identificação de indivíduos com HAS, independentemente do sexo e da faixa etária. De fato, a RCE foi capaz de identificar HAS utilizando o ponto de corte de 0,50 como referência de risco à saúde em amostra representativa, resultados inéditos no Brasil.

Por essa razão e também por ser um marcador de risco cardiometabólico eficaz, prático e de fácil interpretação, não poderíamos negligenciar a propagação do uso rotineiro da RCE como marcador antropométrico de risco à saúde, tanto em pesquisas epidemiológicas como na prática clínica individual e coletiva. A substituição da matriz de associação IMC e CC pela RCE é o elemento de maior relevância na discussão desenvolvida aqui.

Com relação aos pontos de corte da RCE como marcador antropométrico para a população adulta, os resultados não apresentam divergências com a literatura internacional^19^. Na amostra analisada, a utilização dos valores de 0,52 e de 0,54 possibilita a identificação de uma grande parcela de indivíduos com excesso de peso, pois deve classificar corretamente de 84% a 88% dos homens e 83% a 86% das mulheres, indicando ser um valioso marcador antropométrico para o diagnóstico desse distúrbio nutricional.

O uso da RCE é mais vantajoso que a CC por pressupor que, para uma certa estatura, uma determinada quantidade de gordura na região do tronco é aceitável, permitindo assim o estabelecimento de um ponto de corte único e aplicável à população geral, independentemente do sexo e idade^12,20^. Estudos com o objetivo de identificar pontos de corte da RCE e comparar com outras medidas antropométricas de excesso de peso ou discriminadores de fatores de risco cardiometabólicos têm encontrado valores superiores 0,50 como indicativos de risco à saúde^20,21^.

No Brasil, estudo realizado com população específica de adultos e idosos participantes do programa de monitoramento das doenças cardiovasculares e diabetes determinou os pontos de corte para a RCE de 0,52 para homens e 0,53 para mulheres^22^. Estudo realizado por Rodrigues et al.^23^ que teve por objetivo testar a associação entre a RCE e fatores de risco cardiovascular observou os pontos de corte para a RCE, para homens e mulheres, de 0,52 e 0,53 para hipertensão e de 0,53 e 0,54 para síndrome metabólica, concluindo que a RCE foi superior a outras medidas antropométricas na capacidade de identificar tais fatores de risco.

Já é bem documentado na literatura mundial que o sexo e a idade são fatores de risco para o excesso de peso, independentemente do marcador antropométrico utilizado^2,24^. Esses achados são consistentes com os observados no presente estudo, no qual se observa uma tendência de aumento nos valores medianos da RCE com o aumento da idade, com diferenças significativas em função do sexo. A progressão contínua do acúmulo de gordura avaliado pela RCE representa um possível aumento de risco cardiometabólico, conforme estudos recentes^25^.

À semelhança dos inquéritos nacionais^26,27^, este estudo encontrou maior frequência de excesso de peso entre as mulheres. Utilizando a RCE como instrumento de avaliação, prevalências variando de 60% a 68% para adultos e de 86% a 92% para idosos reforçam a evidência de que o excesso de peso é um dos problemas de maior magnitude em saúde pública^28^.

Embora o IMC seja um método internacionalmente aceito para classificação do estado nutricional, sua adoção como padrão único de classificação pode ocasionar avaliações imprecisas e consequentemente diagnósticos errôneos, levando a possíveis intervenções inadequadas no tratamento para o excesso de peso^5^. O pressuposto de que o IMC mede a adiposidade em todas as faixas etárias e com a mesma capacidade pode ser equivocado^29^. As medidas antropométricas que avaliam a concentração de gordura na região abdominal têm apresentado maior capacidade preditiva para os agravos crônicos não transmissíveis e, portanto, são recomendadas na avaliação de saúde dos indivíduos, independente do peso corporal, a exemplo da RCE^12,21^.

Estudos têm comprovado que pessoas com peso normal ou com excesso de peso diagnosticado pelo IMC podem apresentar maior ocorrência de morbidades subestimadas quando apresentam simultaneamente elevação da CC^6,10^. No Brasil, Meller et al.^30^ realizaram estudo com mulheres adultas, sendo constatado que uma em cada quatro mulheres sem excesso de peso apresentavam CC > 80 cm. Resultados semelhantes foram observados em estudo realizado no Maranhão^31^, que encontrou 15,5% de obesidade abdominal em mulheres eutróficas. Assim, para uma avaliação mais apurada de risco à saúde de indivíduos ou de populações, vários pesquisadores têm recomendado a utilização conjunta do IMC e da CC^6^ no intuito de aumentar a acurácia no diagnóstico desse distúrbio nutricional, preditor de inúmeras doenças e agravos à saúde.

Mesmo reconhecendo que a associação de medidas antropométricas pode aumentar a sensibilidade na identificação de risco à saúde, poucos são os estudos realizados no âmbito nacional^31^ que investigaram indivíduos apresentando simultaneamente alteração da CC e excesso de peso. Os dados deste estudo revelam diferenças importantes no diagnóstico de risco entre os sexos, com as mulheres, tanto adultas como idosas, apresentando maiores prevalências, resultados corroborados pelos estudos de Veloso e Silva^31^ e Soares e Barreto^33^.

Estudos internacionais apontam que a combinação do IMC com a CC aumenta as probabilidades de detecção de doenças crônicas não transmissíveis, a saber, hipertensão, diabetes e dislipidemia, em relação à utilização isolada destas medidas^34,35^. Isso posto, devemos nos questionar sobre a praticidade de uso dessas medidas combinadas na prática clínica dos profissionais de saúde. A procura por um marcador simples e que possa triar de forma eficiente um maior número de indivíduos em programas de promoção da saúde e prevenção de doenças e agravos não transmissíveis tem ocupado papel de destaque nas discussões relacionadas ao risco à saúde. Por isso, Ashwell e Gibson^11^ recomendam a substituição da associação entre o IMC e a CC pelo uso rotineiro da RCE, argumentando que esta é uma ferramenta de avaliação de risco primário simples, de fácil interpretação, baixo custo, e que identifica mais pessoas em risco cardiometabólico.

Observou-se neste estudo que 39,6% e 33,3% dos homens e mulheres adultos e 81,8% e 79,1% dos homens e mulheres idosos, respectivamente, categorizados como de baixo risco pela combinação entre IMC e CC apresentaram risco aumentado quando classificados pela RCE e, portanto, poderiam não ser alertados para a necessidade de ações de promoção da saúde e prevenção de doenças e agravos não transmissíveis, à semelhança do constatado por Ashwell e Gibson^11^. Estudo realizado com 36.642 tailandeses adultos também corrobora os resultados aqui apresentados, mostrando que a RCE foi capaz de detectar mais indivíduos em situação de risco cardiometabólico, mesmo quando categorizados como “saudáveis” ou “normais” de acordo com o IMC ou a CC^36^.

A literatura mundial^37^ e brasileira^38^ confirmam elevado poder explicativo tanto para o IMC quanto para a CC nas prevalências de HAS, aumentando ainda mais pela combinação dessas duas medidas^34,35^. Neste estudo, a RCE apresentou habilidades similares na determinação das razões de prevalência para HAS quando comparada à associação dos índices antropométricos IMC e CC, tendo em vista que houve superposição dos intervalos de confiança do risco aumentado avaliado pela RCE com os riscos moderado e elevado avaliados pela matriz de combinação, tanto em adultos como em idosos.

Concluindo, os resultados do presente estudo confirmam dados recentes da literatura, que apontam elevado poder discriminatório da RCE na identificação precoce de indivíduos em risco à saúde, além de apresentar habilidades semelhantes às medidas de adiposidade combinadas na identificação das razões de prevalência de HAS. Assim, a RCE se mostra um importante marcador de risco à saúde, que guarda relação similar com a adiposidade, independente do envelhecimento.

Finalmente, incentivamos a inclusão da RCE na rotina dos serviços e no planejamento das ações em saúde, além da inserção em pesquisas epidemiológicas. A educação em saúde utilizando a mensagem “mantenha sua circunferência da cintura abaixo da metade de sua altura” será mais compreensível e efetiva, em todas as faixas etárias, como atributo de uma vida saudável.
